# More than just an inert dense region

**DOI:** 10.7554/eLife.83076

**Published:** 2022-10-14

**Authors:** Abdou Akkouche, Emilie Brasset

**Affiliations:** 1 iGReD, Université Clermont Auvergne, CNRS, INSERM, Faculté de Médecine Clermont-Ferrand France

**Keywords:** piRNA pathway, heterochromatin formation, HP1 proteins, transposon biology, zinc finger proteins, germline biology, *D. melanogaster*

## Abstract

A newly discovered protein helps define a subset of heterochromatin regions that can silence harmful mobile genetic elements in the genome of fruit flies.

**Related research article** Baumgartner L, Handler D, Platzer SW, Yu C, Duchek P, Brennecke J. 2022. The *Drosophila* ZAD zinc finger protein Kipferl guides Rhino to piRNA clusters. *eLife* **11**:e80067. doi: 10.7554/eLife.80067.

DNA contains all the genetic information necessary to build and maintain an organism. Inside the cell nucleus, DNA is wrapped around histone proteins to form chromatin fibers. Depending on the degree of compaction, the chromatin is classified as euchromatin (less condensed, gene-rich, and more accessible to transcription machinery) or heterochromatin (highly condensed, gene-poor, and transcriptionally silent; Huisinga et al., 2006; Figure 1A).

**Figure 1.**
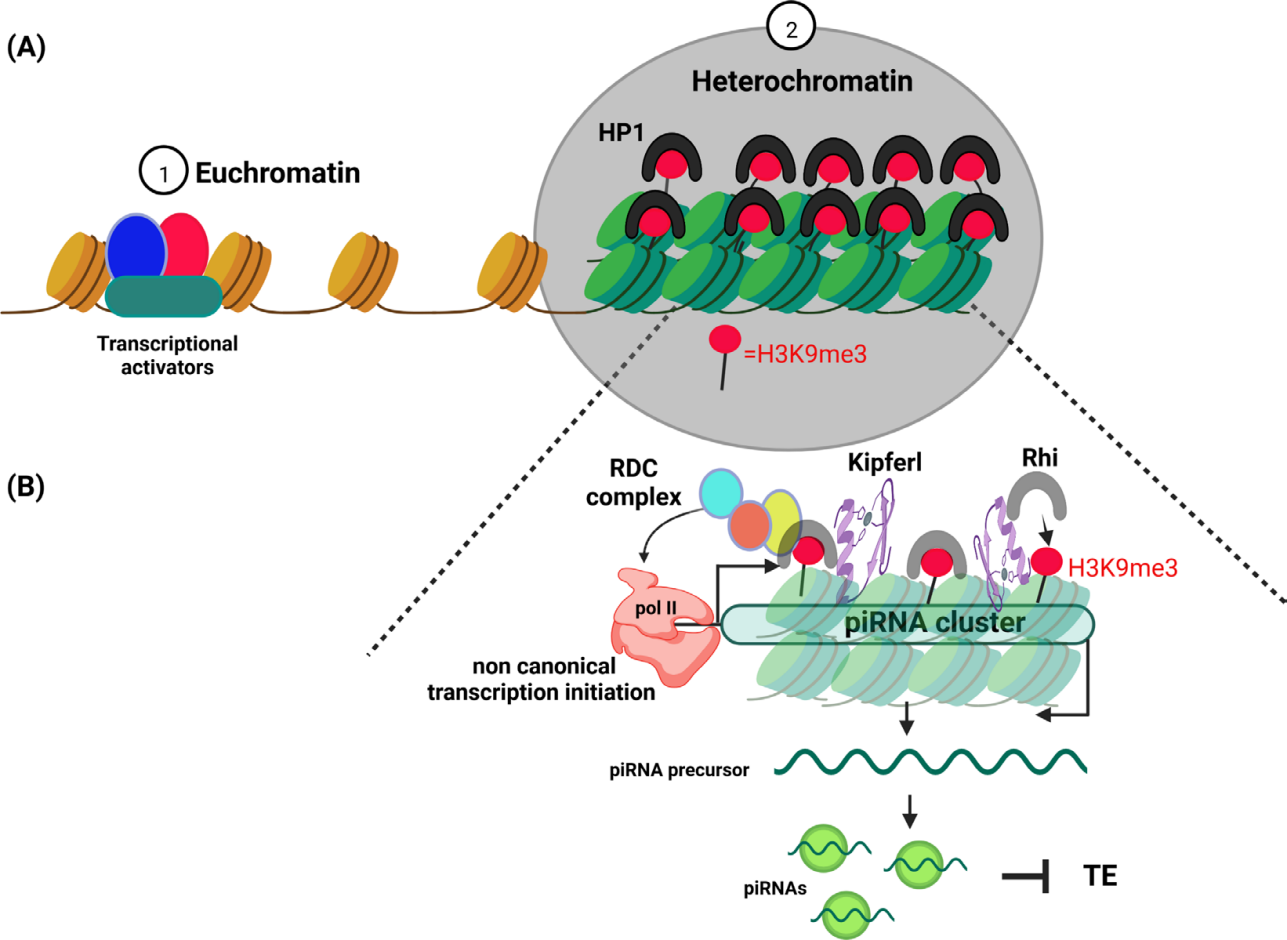
Schematic diagram illustrating euchromatin and heterochromatin compartments. (**A**) Euchromatin (left) is involved in the transcription of DNA into mRNA. It is usually only lightly packed, with the histone proteins it is wrapped around (brown cylinders) spaced far apart so that the DNA can be easily accessed by the cell’s transcription machinery (red, blue and green shapes). Heterochromatin (right) on the other hand, is more densely packed with histone proteins (green cylinders) closely crammed together, making the DNA less accessible. Proteins belonging to the HP1 (Heterochromatin Protein 1) family promote the formation of a condensed chromatin structure by binding to the histone mark H3K9me3 (red circle). (**B**) Heterochromatin has long been thought to be without purpose. However, it has been shown to play an important role in identifying and silencing mobile genetic elements which can disrupt the integrity of the genome. One of the ways cells protect themselves is to transcribe parts of heterochromatin (known as piRNA clusters) that contain these harmful genetic elements. In fruit flies, the DNA-binding protein Kipferl (purple swirl) guides an HP1 protein expressed in germline cells called Rhino (grey horseshoe) to a subset of heterochromatin regions that are enriched in H3K9me3. Rhino then recruits three other proteins to form the RDC complex (made up of Rhino, Deadlock, Cutoff). This complex initiates the transcription of these specific loci with the help of the enzyme RNA polymerase II (peach coloured shape). The resulting transcripts are then processed into piRNAs that guide proteins to silence the mobile genetic elements also called Transposable element (TE).

Heterochromatin had long been considered as genetically inert. However, it turned out to be crucial to the biology of cells and contributes, for example, to chromosome segregation during cell division. It is also important for silencing mobile genetic elements – segments of DNA that can move within the genome or jump into the genome of other cells. Mobile genetic elements can pose a real threat to the integrity of the genome, and consequently, several defence mechanisms have evolved to identify them and prevent them from multiplying.

Some heterochromatic loci, formed of remnants of mobile genetic elements, are at the center of these defence mechanisms. These parts of the genome, also known as piRNA clusters, are often compared to a ‘memory system’. Despite being heterochromatic and usually silent, they are transcribed and processed into small non-coding RNAs. These RNAs are called PIWI-interacting RNAs, or piRNAs for short, because they are loaded by proteins of the PIWI family (Figure 1B). They use sequence complementarity to recognize mobile genetic elements residing in other parts of the genome, and then silence them (Brennecke et al., 2007).

In many eukaryotes, the molecular feature defining heterochromatin is the enrichment of a specific histone mark called H3K9me3. This mark is recognized by proteins belonging to the HP1 (Heterochromatin Protein 1) family, which help to pack DNA into its condense structure (Bannister et al., 2001; Lachner et al., 2001). A member of this family, known as Rhino, facilitates the transcription of piRNA clusters in the germline of fruit flies. Although Rhino displays specific affinity for H3K9me3, it only binds to a subset of heterochromatin regions that contain this histone mark. So far, it was unclear how Rhino is guided to these specific parts of the genome (Mohn et al., 2014). Now, in eLife, Julius Brennecke and colleagues – including Lisa Baumgartner as first author – report the results of experiments that clarify this process (Baumgartner et al., 2022).

The team (who are based at the Vienna BioCenter and the Institute of Molecular Biotechnology of the Austrian Academy of Sciences) used a combination of genetic, genomic and imaging approaches to study Rhino in the germline of female fruit flies. The experiments revealed that Rhino interacts with a DNA-binding protein, which Baumgartner et al. named Kipferl. Indeed, a depletion of this protein in the ovaries of fruit flies leads to a broad redistribution and concentration of Rhino at the nuclear periphery in a form that evokes the shape of a Kipferl, an Austrian croissant.

They found that both Kipferl and Rhino are bound at many piRNA source loci, suggesting that Kipferl may act to identify the heterochromatin regions to which Rhino must bind. In the absence of Kipferl, Rhino is sequestered to another part of the genome and its binding to some piRNA clusters is lost, despite the presence of H3K9me3 marks. As a result, piRNA production in ovaries lacking Kipferl is reduced, several mobile elements are reactivated, and the females are less fertile than flies expressing Kipferl.

Kipferl is a DNA-binding protein that specifically binds to DNA sequences that are rich in the guanine nucleotide. This study suggests for the first time that such DNA sequences could help to attract Kipferl, which then recruits Rhino participate in defining piRNA clusters. However, while Kipferl guides and stabilizes Rhino to some chromatin domains enriched in H3K9me3 to convert them into piRNA clusters, some Rhino-dependent piRNA clusters do not need Kipferl (Figure 1B).

The study of Baumgartner et al. also suggests that additional factors help guide Rhino to piRNA clusters during early oogenesis, as Kipferl is not expressed during this developmental stage. Furthermore, piRNAs provided by the mothers may also help to recruit Rhino to specific heterochromatin regions in the embryo (Akkouche et al., 2017; Le Thomas et al., 2014). However, the relative contribution of maternally deposited piRNAs and Kipferl in recruiting Rhino to specify piRNA clusters during embryogenesis requires future investigations.

The study of Baumgartner et al. shows that the relationship between Rhino, Kipferl and DNA is complex, and their elegant dissection of the role of Kipferl provides substantial new insight into how piRNA clusters are defined in the genome.
